# The induced membrane technique: A therapeutic option for managing bone defects in the upper extremity: Case series for 7 patients

**DOI:** 10.1016/j.amsu.2022.104533

**Published:** 2022-09-02

**Authors:** Amine El Farhaoui, Kamal Benalia, Adnane Lachkar, Najib abdeljaouad, Hicham Yacoubi

**Affiliations:** a Faculty of Medicine and Pharmacy, Mohammed I^st^ University, Oujda, Morocco; b Department of Traumatology, Orthopedic Mohammed VI University Hospital Mohammed I University, Oujda, Morocco

**Keywords:** Induced membrane technique, Bone loss, Graft, Biological cement, Upper limb

## Abstract

**Introduction:**

The reconstruction of bone defects of tumoral, infectious or traumatic origin of the limbs remains a major therapeutic challenge for the orthopedic surgeon and the patient, in terms of anatomical and functional results.

**Cases presentation:**

We report the case of 7 patients who underwent induced membrane bone reconstruction of the upper extremity, 5 patients with initial injury to the forearm, and 2 of our patients, to the humerus. In terms of function, the range of prono-supination was 125°, the range of wrist flexion-extension was 165°, and the range of elbow mobility was 170°. All patients achieved union at the time of the last follow-up. Two patients achieved union at 6 months, one patient at 5 months, one patient at 4 months, and three patients at 3 months.

**Discussion:**

The induced membrane (IM) technique has been used for more than 30 years, and it's more and more widely accepted all over the world, as a simple and effective technique for reconstruction of segmental bone defects. The technique comprises 2 surgical stages, The first step involves the total excision of infected and non-viable lesions both bone and soft tissue until tissue with optimal vascularization "Paprika sign", then the strict instrumental stabilization of the skeleton and the realization of a covering flap if necessary, depending on the site of the initial injury initial lesion and the extent of the resection.

**Conclusion:**

The technique of induced membrane has proven its effectiveness in the management of bone loss.

## Highlights

• The induced membrane (IM) technique has been used for more than 30 years, and it's more and more widely accepted all over the world, as a simple and effective technique for reconstruction of segmental bone defects.
• Short-term functional outcome was satisfactory in all our patients.
• Managing bone defects in the long bones of the upper extremity is more problematic.

## 1 Introduction

The reconstruction of bone defects of tumoral, infectious or traumatic origin of the limbs remains a major therapeutic challenge for the orthopedic surgeon and the patient, in terms of anatomical and functional results [1]. Multiple treatment options are available, including autograft with non-vascularized fibula or cancellous bone graft, vascularized bone graft and bone transport by Ilizarov technique, even massive allograft transplantation and recently tissue engineering techniques [2], all these therapeutic modalities have proven their effectiveness but some are very demanding as a result their indication remains restricted due to lack of means.

Managing bone defects in the long bones of the upper extremity is more problematic, for instance single-stage plate fixation and autologous bone grafting can be useful in the treatment of forearm nonunions [3,4] but have been associated with poor outcomes when the defect size exceeds 5 cm due to a high probability of graft resorption[5,6].

Overall, there have been relatively few reports on the use of the Masquelet technique in the upper extremity [7]. [8]. This work summarizes our department's experience with the Masquelet technique for reconstruction of bone loss long bones of the upper limb.

## 2 Cases presentation

### 2.1 - Patient 1

**Fig. 1:**
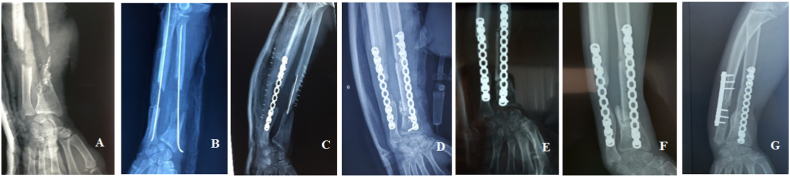
A. X-ray on admission: fractures of the middle 1/3-lower 1/3 junction of the ulna and radius with PSO/B. Control X-ray after Metaizeau pinning/C. Rx after cancellous grafting of the ulna and placement of the cement for the radius/D. Postoperative T2 Masquelet Rx/E. Rx at 5 months/F. Rx at 7 months/G. Rx after shortening osteotomy.

The first patient, 42 years old, had an open fracture of both forearm bones classified as Cauchoix and Duparc stage II, and Gustillo stage IIIc with total section of the radial artery, rupture of the flexor pollicis longus and slight contusion of the median nerve; the standard radiograph showed acute loss of bone substance "Karger III" of the radius and "Karger II" of the ulna. The patient initially underwent centromedullary pinning of the two bones of the forearm with vascular and tendon repair; then, after two weeks, he received an immediate cancellous graft for the ulna and a 1st stage Masquelet for the radius bone defect fixed by a centromedullary pin. The patient was readmitted after 6 weeks for the 2nd stage of Masquelet using a non-vascularized fibular graft fixed with a screwed plate. The 6-month follow-up X-rays showed good osseointegration of the two cancellous grafts (iliac ulnar and fibular radial) with no signs of resorption; clinically, there was a radial tilt of the left hand following ulnar lengthening and no signs of infection; functionally, there was limited pronosupination and recovery of post-traumatic median nerve palsy. Subsequently, the patient underwent an ulna shortening osteotomy. Recoil at 18 months, pronation at 85°, supination at 90°, slight limitation of wrist flexion (Fig. 1).

### 2.2 - Patient 2

A patient of 18 years old, victim of a traffic accident (car rollover) causing him an open fracture of both forearm bones (stage III CD, stage IIIc Gustillo) with total section of the radial artery, the radiograph showed a bifocal fracture of the ulna with spiral fracture of the radius, The first step of the therapeutic management was debridement of the non-viable contaminated tissue and reconstruction of his radial artery with a graft taken from the saphenous vein with placement of a radial external fixator and a minimal olecranon pinning for the ulna. A few weeks later, an autologous skin graft was placed, and later he had the pins removed and plates placed for the two bones of the forearm. 15 months later the patient presented to the consultation with radial tilt and limitations of the wrist joint amplitudes, notably prono-suppination, standard radiography showed consolidation of the ulna and pseudarthrosis of the radius with a 2 cm "Karger II" defect; the team's choice was directed towards bone reconstruction by the IM technique; in the first stage a gentamycin cement spacer was placed to fill this defect fixed by two pins. The second stage of Masquelet was performed after 6 weeks. The evolution was favorable with the formation of a bone callus leading to consolidation of the fracture site. The patient had benefited from a therapeutic rehabilitation protocol to restore limb function. The final result was satisfactory with good recovery of joint range of motion at the last follow-up.

### 2.3 - Patient 3

**Fig. 2:**
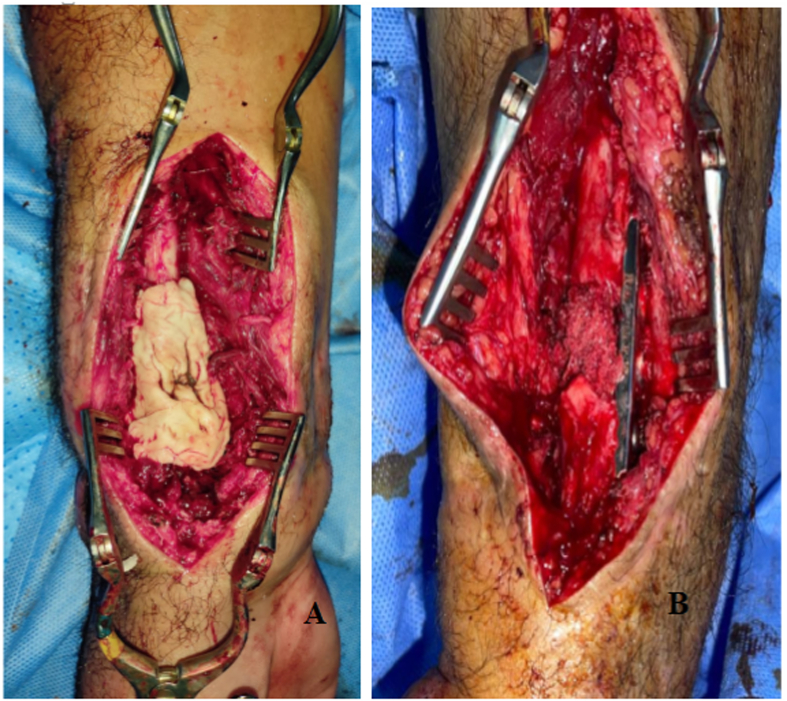
Intraoperative image "patient 3": A. placement of biologic cement (T1)/B. placement of graft and plate stabilization.

A 28-year-old patient had an open fracture of both forearms (classified as stage II CD, stage III Gustilo), with section of the flexor pollicis longus of the thumb, the deep and superficial flexors of the 3rd, 4th, and 5th fingers, and total section of the radial artery. The radiograph revealed a comminuted fracture of the lower extremity of the radius and the ulna; The patient underwent repair of these arterial and tendinous lesions, followed by a centromedullary pinning of the ulna, an external fixator of the upper limb with 2 plugs on the radius and 2 plugs on the 2nd and 3rd metacarpals, and the placement of two styloid pins; After 3 months, the patient presented to the clinic with local inflammatory signs and pus from the surgical site, the biological assessment showed a CRP of 95 and 10,500 WBC, the X-ray showed that the comminuted fracture had not consolidated, which is why the patient was taken to the operating room, where he underwent lavage, trimming and placement of biological cement with gentamicin, and fixation with centromedullary wires A suitable antibiotic therapy was then administered, and then the second stage of Masquelet was performed after 2 months using a cortico-cancellous graft of the anterior iliac crest fixed by screwed plate. The evolution was marked by the recovery of the joint amplitudes (pronation at 80°, supination at 70°, flexion of the thumb, flexion and extension of the fingers preserved) and the constitution of a bony callus (Fig. 2).

### 2.4 - Patient 4

A 27 years old patient had a simple fracture of the humeral diaphysis treated with a screwed plate. After 3 months, the patient presented with pain and inflammatory signs at the level of the approach, the radiograph showed a non-union of his fracture; we performed the first stage of Masquelet with external fixation, and then the patient was put on Imipenem and Ciprofloxacin because of the detection of Klebsiella in the sample taken intraoperatively. After a close follow-up of his skin condition and his biological check-up, the second stage of Masquelet was performed at 10 weeks with the use of a cortico-cancellous graft stabilized by a screw. After 6 months, the patient had satisfactory elbow mobility with no signs of radial paralysis: Good consolidation and incorporation of the graft.

### 2.5 Patient 5

57 year old female patient, who suffered a closed arm trauma resulting in a fracture of the humeral palette treated with an external plate. After 7 months she consulted for pain in the limb, paresis of the radial nerve and skin fistulization opposite the surgical site; the inflammatory workup was found to be disturbed, the EMG showed axonotmesis-type nerve damage with reinnervation potential, and the radiographs showed an inter-fragmentary gap with no signs of consolidation; After debridement of the non-viable bone tissue, we filled the defect (4 cm "Karger III") with a cement spacer and fixed it with two ulnohumeral pins, thus performing a neurolysis of the radial nerve; Subsequently, the osteosynthesis material was replaced by two plates on either side of the paddle with an osteotomy of the olecranon fixed by pinning and bracing. The patient was lost to follow-up and returned after 22 months with residual pain; the X-ray showed a break in the plate. The second stage of Masquelet was performed with fixation of the graft by two plates then she had benefited from a therapeutic rehabilitation protocol to restore the function of the limb. The final result was satisfactory with good recovery of joint amplitudes at the last follow-up.

### 2.6 - Patients 6 and 7

These are two patients who presented with sepsis on the material at 5 and 3 weeks respectively after the operation. The first had a comminuted fracture of the lower extremity of the radius associated with a distal radio-ulnar dislocation, initially treated with a console plate and stabilization of the radio-ulnar with a pin, while the second had a simple fracture at the junction of the middle and lower thirds of the radius associated to a radial nerve paralysis, treated with a plate. Both patients benefited from trimming and bacteriological samples, were put on appropriate antibiotics, and then the first stage of Masquelet with external bone fixation was performed. After clinical and biological cooling of the infection, the second stage was performed at 6 weeks with stabilization of the graft by screwed plate.

**Fig. 3:**
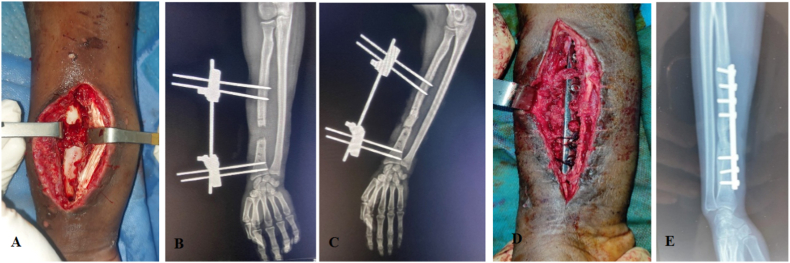
Intraoperative and radiological images of a patient with sepsis on material "patient 7″ **A**. Bone defect after debridement of non-viable bone tissue/**B**. Post-op Rx after trimming/**C**. Post-op Rx of T1/D. Peroperative image of T2/**E**. Rx after 3 months of T2.

At 3 months, both of patients presented with no signs of infection in the area of the scar, and recovery of her articular amplitudes, radiologically, good integration of the cancellous graft and appearance of bone bridges in the area. However, The second patient remains affected by radial nerve paralysis (Fig. 3).

## 3 Discussion

The induced membrane (IM) technique has been used for more than 30 years, and it's more and more widely accepted all over the world, as a simple and effective technique for reconstruction of segmental bone defects [9].

The initial clinical experience with the induced membrane technique was in post-traumatic septic pseudarthrosis of the leg. Progressively, the indications were extended to other etiologies of bone loss:

• In post-trauma: limb salvage in the case of complex fractures, treatment of non-union
• In tumor pathology: bone reconstruction after tumor resection, 12.9% in Masquelet's original series [10], and 22.9% of cases reported by Zappaterra in France [11].
• In infectious pathology: bone loss resulting from chronic osteitis, or osteomyelitis sequestration [2].

In our series, one patient was treated for post-traumatic bone loss resulting from open and complex fracture, four patients for following infected or uninfected nonunion and two patients had an osteitis due to infection on osteosyhthesis material.

The longest bone loss described in the literature, treated by the IM technique, was of the order of 35 cm [12], classified as IV according to the Karger classification, but in no case did the length of the SOP influence consolidation [10]. In similar study to our study the median bony defect at the time of definitive reconstruction was 5,3 cm (2,5–8 cm) [11]. In our series, the average was 3.64 cm (1.5–8 cm).

The technique comprises 2 surgical stages, The first step involves the total excision of infected and non-viable lesions both bone and soft tissue until tissue with optimal vascularization "Paprika sign", then the strict instrumental stabilization of the skeleton and the realization of a covering flap if necessary, depending on the site of the initial injury initial lesion and the extent of the resection. Once the debridement and trimming are completed, an acrylic cement spacer is inserted into the bone loss, after multiple samples have been taken for multiple bacteriological samples (at least 3) [2]. The cement should encase the bone ends, and pushed intramedullary, until total stability of the implant is achieved to construct a continuous membrane of excellent quality and of sufficient thickness to envelop the subsequent bone graft [13].

We used an antibiotic cement: Gentamycin-PMMA in cylinder. A recent study has been published by Nau [14] in Germany, evaluate the properties of the membrane obtained in contact with three antibiotic cements. The results showed a thicker membrane thicker with a significant presence of immature vessels in the case of gentamycin cement, without cement, but without being able to evaluate the osteogenic quality of this induced membrane. As for bone fixation during T1, we used the external fixator and centromedullary wires as means of osteosynthesis depending on the initial skin condition, the type of fracture and the location of the bone defect.

The second surgical step is performed at least after 6–8 weeks of the first step, however this term is not however this term is not strict because it must respect the healing of the soft parts and the control of any and the control of any possible infection present, based on clinical and biological criteria or bacterial and biological criteria or bacteriological samples at the slightest doubt, however a delay of several months or even several years does not seem to be detrimental according to the literature and does not quality of consolidation, because the bone graft in the second stage can be considered a second stage can be considered as a foreign body likely to reactivate the biological properties of the biological properties of the membrane[11]. [13].

This step consists of incising the induced neoformed membrane with a cold blade, removing the cement spacer, cleaning the residual cavity while preserving the membrane, decorticating the bone ends, repermeabilizing the medullary canals, and petalizing the cortical ends, to facilitate the placement of the bone graft and thus ensure good consolidation [15].

We used cancellous bone and cortico-cancellous bone as a graft to fill the cavity chamber taken from the patient's anterior iliac crests. The cancellous bone must be broken up into small fragments not exceeding 2–3 mm, ensuring that the membrane is completely filled in to avoid the gravitational effect. Eventually, the association of a non-vascularized multiperforated fibular segment was used for a patient who had extensive radius substance loss following an acute post-traumatic PSO, thus allowing primary mechanical stability. To facilitate revascularization of the graft by the membrane, a rigid mounting must be performed, we used the screw plate for all our patients which is recommended by Masquelet 1 and described in similar studies [11]. [16].

The average duration of follow-up was 3.2 years. All other patients had reached a final evaluation. No additional procedures to stimulate bone union were required (Table 1).

**Table 1:** Results of the Masquelet Technique in our case series.

patient	Affected Bone	Defect Type	Size of Defect (cm)	Time Between Masquelet Stages 1 and 2 (weeks)	Type of Graft Used in Stage 2	Time to Radiographic Union (months)
1	Midshaft Radius + ulna	Acute open fracture	9 cm (ulna)	6	Fibular graft	6
2	Midshaft Radius	uninfected non-union	2 cm	6	cancellous graft	4
3	Distal Radius + ulna	Infected non-union	1.5 cm	8	cortico-cancellous graft	3
4	Midshaft Humerus	Infected non-union	3 cm	10	cortico-cancellous graft	6
5	distal humerus	Infected non-union	5 cm	22	cortico-cancellous graft	5
6	Distal radius	Osteitis	2 cm	6	cortico-cancellous graft	3
7	Midshaft radius	Osteitis	3 cm	6	cortico-cancellous graft	3

In terms of function, the prono-supination range was 125° (Pr.70-Sp.85°), the wrist flexion-extension range was 165° (Fl.75°-Ex.87°) and the elbow mobility range was 170°. All patients achieved union by the time of the latest follow-up. Two patients achieved union by 6 months, one patients by 5 month, one patient by 4 month and three patients by 3 months.

One patient underwent additional surgery, ulnar splinting osteotomy. Two patients still had moderate forearm pain on final evaluation. None of the patients had donor site complications.

The PROCESS guildlines were used in the writing of this paper [17].

## 4 Conclusion

The technique of induced membrane has proven its effectiveness in the management of bone loss, so we need to have more studies in the direction of the practice of this method, especially when it comes to the loss of the upper limb.

## Ethical approval

The ethical committee approval was not required give the article type (cases series). However, the written consent to publish the clinical data of the patients was given and is available to check by the handling editor if needed.

## Sources of funding

None.

## Author contribution

AMINE EL FARHAOUI: study concept or design, data collection, data analysis or interpretation, writing the paper, KAMAL BENALIA study concept or design, data collection, data analysis or interpretation, writing the paper, ADNANE LACHKAR: supervision and data validation, NAJIB ABDELJAOUAD: supervision and data validation, HICHAM YACOUBI: supervision and data validation.

## Registration of research studies

This is not an original research project involving human participants in an interventional or an observational study but a cases series. This registration is was not required.

## Guarantor

Amine El Farhaoui.

Kamal Benalia.

## Consent for publication

Written informed Consent was obtained from the patients for publication of this case report and accompanying images. A copy of the written consent is available for review by the Editor-in-Chief of this journal on request.

## Provenance and peer review

Not commissioned, externally peer reviewed.

## Declaration of competing interest

The authors declare no conflicts of interest.
